# PtrbZIP12 improves drought resistance in *Populus trichocarpa* by directly targeting PtrDHN and PtrPOD

**DOI:** 10.1093/hr/uhag034

**Published:** 2026-02-05

**Authors:** Meiqi Zhou, Yilin Wang, Kim Lien Phan Thi, Yao Chi, Xu Li, Yang Li, Chao Wang

**Affiliations:** State Key Laboratory of Tree Genetics and Breeding, Northeast Forestry University, Harbin 150040, China; State Key Laboratory of Tree Genetics and Breeding, Northeast Forestry University, Harbin 150040, China; State Key Laboratory of Tree Genetics and Breeding, Northeast Forestry University, Harbin 150040, China; State Key Laboratory of Tree Genetics and Breeding, Northeast Forestry University, Harbin 150040, China; State Key Laboratory of Tree Genetics and Breeding, Northeast Forestry University, Harbin 150040, China; State Key Laboratory of Tree Genetics and Breeding, Northeast Forestry University, Harbin 150040, China; State Key Laboratory of Tree Genetics and Breeding, Northeast Forestry University, Harbin 150040, China

## Abstract

This research examines how the basic leucine zipper (bZIP) transcription factor (TF) influences drought stress responses in tree species, emphasizing its related regulatory pathways, and thus offering a theoretical framework for understanding drought response mechanisms regulated by the bZIP TF family. Specifically, we characterized the functional role of the S subfamily bZIP gene, *PtrbZIP12*, from *Populus trichocarpa*, by developing transgenic poplars that either overexpressed or knocked down *PtrbZIP12*. The findings indicated that *PtrbZIP12* markedly improved drought tolerance in transgenic plants by facilitating reactive oxygen species scavenging, enhancing proline biosynthesis, and reducing plasma membrane peroxidation and cell death. To pinpoint *PtrbZIP12*’s downstream targets, RNA sequencing was performed, followed by chromatin immunoprecipitation-PCR (ChIP-PCR), yeast one-hybrid, and dual-luciferase assays. These analyses confirmed that *PtrbZIP12* binds directly to the promoters of *PtrDHN* (dehydrin) and *PtrPOD* (peroxidase), leading to the activation of their expression. Transgenic poplars overexpressing (OE) *PtrDHN* or *PtrPOD* were subsequently generated, and similar to *PtrbZIP12*, their OE conferred enhanced drought tolerance. Moreover, coexpression of PtrbZIP12 with PtrbZIP3 further elevated *PtrDHN* transcript levels, resulting in improved drought resilience in the *PtrbZIP12* transgenic lines. Moreover, phosphorylation was identified as a key factor in boosting PtrbZIP12-mediated transcriptional regulation of *PtrPOD* and *PtrDHN*, underscoring the significance of posttranslational modification in plant drought stress responses.

## Introduction

Amid global climate change, recent years have seen a notable rise in both the occurrence and intensity of extreme weather events, with droughts being especially prevalent [1]. These droughts exert profound and widespread impacts on forest growth, posing serious threats to the structure, stability, and functioning of forest ecosystems. Reduced soil moisture under drought stress hampers tree growth, diminishes productivity, and can ultimately lead to tree mortality. Such outcomes not only disrupt timber production but also compromise critical ecological services, including global carbon sequestration and biodiversity conservation [2, 3].

Under drought stress, plants have developed a range of complex biochemical and physiological adaptation strategies that allow them to endure unfavorable conditions [4]. A central challenge during drought is the disruption of reactive oxygen species (ROS) homeostasis. In plant cells, drought stress disrupts the balance between ROS scavenging and production, causing an abnormal buildup of these reactive molecules. Elevated ROS levels induce oxidative stress, causing significant damage to membrane proteins, lipids, and other vital cellular structures [5, 6]. Interestingly, when present at low concentrations, ROS act as essential signaling molecules that influence plant development, stress adaptation, immune responses, and growth [7, 8]. To counter oxidative stress and reestablish ROS equilibrium, plants utilize a complex antioxidant defense network composed of enzymatic elements, such as peroxidase (POD), superoxide dismutase (SOD), and catalase (CAT), alongside nonenzymatic antioxidants, including glutathione, carotenoids, and ascorbic acid, all functioning together to sustain ROS homeostasis [9–11].

Plants have established, via long-term evolution, a sophisticated stress response network that integrates hormone regulation [12], osmotic adjustment, transcription factor (TF) regulation, and signal transduction pathways [13]. Among these, TFs serve as pivotal regulatory hubs that mediate plant adaptation to environmental stress by binding to specific promoter regions of target genes [14]. The basic leucine zipper (bZIP) family constitutes one of the most extensive and widely distributed classes of plant TFs. The bZIP protein can specifically bind to the promoter motif containing ACGT through its conserved leucine zipper domain, thereby regulating the transcription of target genes [15]. Based on both sequence homology and functional diversity, the bZIP family is categorized into subfamilies A–I and S [16–18]. Each subgroup contributes to distinct aspects of plant development, growth, and responses to environmental cues, including sugar signaling [19, 20], abscisic acid (ABA) signaling [21], light signaling [22, 23], abiotic and biotic stress responses [24, 25], biosynthesis of chlorophyll and anthocyanins [26, 27], and defense against pathogens. For instance, subfamily A predominantly participates in drought tolerance and ABA signaling [28], whereas subfamily S members are commonly induced under osmotic stress conditions, leading to elevated expression levels [29]. Notably, bZIP TFs function as homo- or heterodimers through their leucine zipper domains, and this dimerization capability is critical to the diversity of their regulatory functions [30]. Subfamily members can interact within or across subfamilies to form dimeric complexes with distinct DNA-binding specificities and transcriptional activities, thereby enabling fine-tuned regulation of complex gene expression networks [31]. Specifically, S subfamily members play pivotal roles in abiotic stress responses, particularly drought and osmotic stress, owing not only to the strong induction of their expression by such stimuli but also to their capacity to form with other such members of the bZIP subfamilies [32]. This interaction-based mechanism is believed to integrate multiple stress signaling pathways and amplify stress-responsive transcriptional outputs, granting the S subfamily a unique and essential role in orchestrating integrated stress resilience. Nevertheless, the precise regulatory mechanisms that govern the formation, specificity, and functional consequences of these heterodimers, particularly those involving S subfamily proteins, remain poorly understood. Thus, advancing our understanding of the composition, regulatory dynamics, and functional specificity of S subfamily-containing heterodimers is essential for unveiling new insights into the intricate transcriptional networks that underpin plant adaptation to environmental challenges.

Protein phosphorylation is a critical regulatory mechanism in ROS signaling pathways and plays an essential role in modulating the activity of TFs [33, 34]. bZIP TFs can be phosphorylated by multiple kinases, including CDPKs, SnRKs, and MAPKs. These posttranslational modifications significantly affect their transcriptional activity, stability, and ability to interact with other regulatory proteins, enabling precise regulation of downstream stress-responsive gene expression [35–37]. For instance, the phosphorylation of serine/threonine residues within the basic region of bZIP proteins by SnRKs is a pivotal step required for their functional activation [38]. While numerous studies have highlighted the involvement of the bZIP TF family in enhancing plant stress tolerance, the detailed regulatory mechanisms governing their phosphorylation, the complexity of their interaction networks, and the sequence of regulatory cascade events during stress responses remain largely elusive.

This study investigated the role of bZIP TFs in *Populus trichocarpa* under drought stress, leading to the identification of a bZIP gene, *PtrbZIP12*, which was markedly induced during drought and classified within the S subfamily. Functional evaluation showed that OE *PtrbZIP12* markedly improved drought tolerance in transgenic poplars. Mechanistic investigations revealed that the PtrbZIP12 protein directly binds to the promoters of its downstream target genes, *PtrPOD* and *PtrDHN*, thereby activating their transcription. This activation enhanced the plants’ ability to scavenge ROS, minimized cell death and membrane lipid peroxidation, and ultimately strengthened their drought resilience. Furthermore, PtrbZIP12 was found to interact with PtrbZIP3, an A subfamily member also induced by drought, and their coexpression synergistically elevated *PtrDHN* transcript levels. Finally, phosphorylation of PtrbZIP12 by PtrSnRK2 kinase was shown to further augment its transcriptional activation of *PtrDHN* and *PtrPOD*, highlighting the importance of posttranslational modification in fine-tuning drought-responsive gene expression.

## Results

### Drought stress induced expression of *PtrbZIP12* gene in *P. trichocarpa*

To identify the key bZIP TFs involved in the response to drought stress, based on the previous drought transcriptome data from the laboratory, we analyzed the FPKM values of poplar plants under drought treatment (Table S1). Among the bZIP genes of the S subfamily, *PtrbZIP12* has the highest upregulated expression multiple under drought conditions (Fig. S1), indicating that it may play a core role in drought adaptation. Therefore, we chose *PtrbZIP12* for further research on its functions and mechanisms.

**Figure 1:**
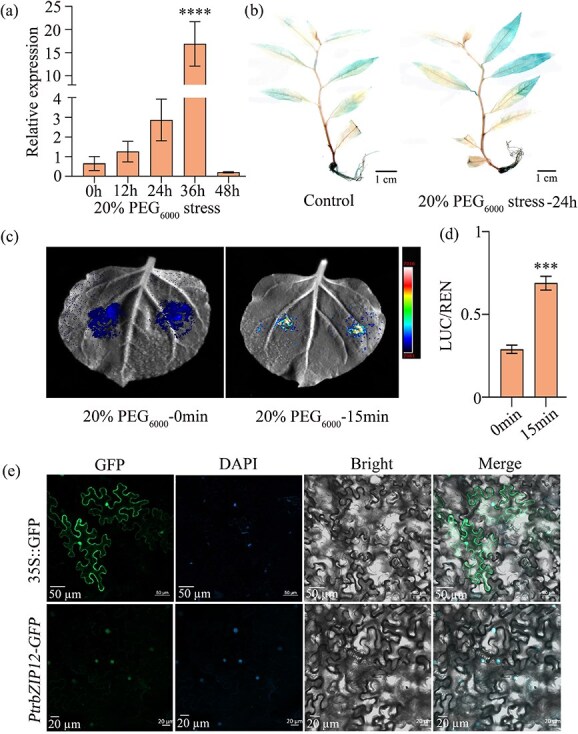
Expression characteristics and subcellular localization of PtrbZIP12. (a) Transcript abundance of *PtrbZIP12* under 20% PEG_6000_ treatment. (b) GUS staining analysis of *PtrbZIP12* promoter activity in transgenic poplar leaves. (c) Luminescence intensity mapping in *N. benthamiana* leaves transiently transformed with *Agrobacterium*. (d) Quantitative assessment of *PtrbZIP12* promoter activity using LUC reporter assays. (e) Subcellular localization of the PtrbZIP12 protein in *N. benthamiana* epidermal cells, with panels displaying GFP fluorescence, bright-field imaging, DAPI nuclear staining, and a merge composite image for spatial reference. (a and d) Data are presented as means ± SD (*n* ≥ 3). Asterisks denote significant differences versus the control (one-way ANOVA with Tukey’s test) (^*^P < 0.05, ^**^P < 0.01, ^***^P < 0.001).

To systematically characterize the drought stress response of the *PtrbZIP12* gene, we examined its expression profile under PEG_6000_-induced osmotic stress using qRT-PCR. The results revealed a progressive increase in *PtrbZIP12* transcript abundance with prolonged stress exposure relative to the control group (Fig. 1a). To further investigate promoter responsiveness, transgenic poplar plants (*ProPtrbZIP12*::GUS) were generated by fusing a 1.5 kb *PtrbZIP12* promoter fragment to the β-glucuronidase (GUS) reporter gene. After drought treatment, GUS staining revealed a marked rise in reporter expression within the leaves (Fig. 1b). Moreover, the *PtrbZIP12* promoter fused to the luciferase (LUC) reporter gene was transiently introduced into *Nicotiana benthamiana* leaves via *Agrobacterium*-mediated transformation. After drought stress, luminescence intensity from the LUC reporter was approximately threefold higher than that of the control (Fig. 1c and d). Taken together, these results demonstrate that *PtrbZIP12* exhibits strong induction in response to drought stress and that its promoter displays potent transcriptional activity associated with drought responsiveness.

For subcellular localization analysis of PtrbZIP12, a PtrbZIP12-GFP fusion construct was transiently transferred into *N. benthamiana* epidermal cells. In cells carrying the empty vector control (pBI121-GFP), GFP fluorescence appeared evenly dispersed across the nucleus and cytoplasm. In contrast, cells expressing the PtrbZIP12-GFP fusion protein (pBI121-PtrbZIP12-GFP) displayed green fluorescence exclusively in the nucleus (Fig. 1e), confirming that PtrbZIP12 is localized to the nucleus.

### *PtrbZIP12* confers drought tolerance to transgenic plants

**Figure 2:**
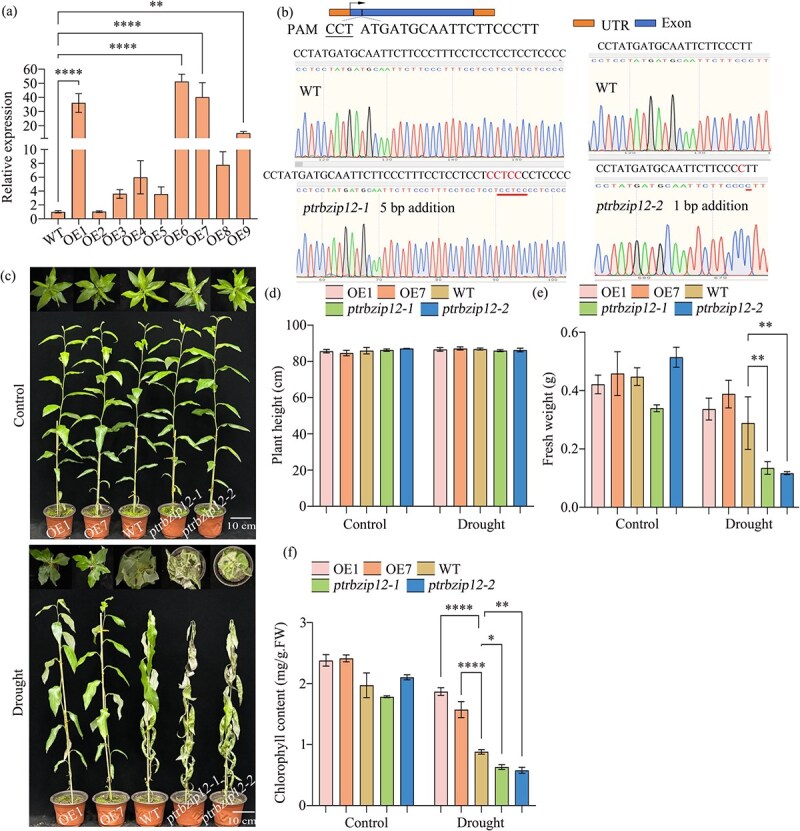
Functional characterization of *PtrbZIP12* in conferring drought tolerance. (a) Relative *PtrbZIP12* expression levels in OE lines were quantified, with transcript values log_2_-transformed relative to WT plants (set as 0), where positive values suggest upregulation. (b) CRISPR/Cas9-mediated mutations in *PtrbZIP12* were confirmed through Sanger sequencing, with annotated target sequences and nucleotide insertions. (c) Phenotypic differences among OE, WT, and *ptrbzip12* lines were assessed under both well-watered (Control) and drought-stressed conditions, the latter induced by a 7-day water-withholding treatment; the scale bar represents 10 cm. (d) Comparative analysis of plant heights. (e) Fresh weight measurements and (f) chlorophyll (Chl) content evaluations were conducted. Poplar growth phenotypes were monitored under normal conditions and drought stress for 90 days, with control plants receiving regular watering and drought-stressed plants subjected to 7 days without water. Each experimental replicate consisted of 5–10 plantlets. Data are presented as means ± SD (*n* ≥ 3). Asterisks denote significant differences versus the control (one-way ANOVA with Tukey’s test) (^*^P < 0.05, ^**^P < 0.01, ^***^P < 0.001, ^****^P < 0.0001).

To clarify *PtrbZIP12*’s function in drought response, we generated CRISPR/Cas9-mediated knockout lines together with transgenic lines that overexpressed *PtrbZIP12*. qRT-PCR analysis verified successful overexpression (OE) of *PtrbZIP12* in transgenic plants (Fig. 2a), which were designated as OE1–OE9. DNA sequencing of the CRISPR-edited plants revealed two distinct mutation events (Fig. 2b), designated *ptrbzip12-1* and *ptrbzip12-2*, both of which altered the native amino acid sequence of *PtrbZIP12* and led to premature translation termination. For subsequent functional analyses, two OE lines (OE1 and OE7) exhibiting similarly high fold changes in expression, along with two CRISPR lines (*ptrbzip12-1* and *ptrbzip12*-*2*), were selected.

Under normal growth conditions, neither the *PtrbZIP12* OE lines nor the *PtrbZIP12* mutant lines showed any significant growth differences compared with wild-type (WT) poplars. However, when subjected to drought stress, the *PtrbZIP12* mutant plants exhibited a distinctly drought-sensitive phenotype (Fig. 2c), characterized by pronounced leaf damage and desiccation compared with the WT plants and OE lines. In contrast, under identical conditions, the OE lines exhibited only slight leaf damage. Moreover, the OE transgenic plants demonstrated greater plant height and fresh weight than the WT, whereas the mutants had noticeable reductions in both parameters (Fig. 2d and e). Similarly, the chlorophyll content was markedly higher in the OE lines and markedly lower in the mutants relative to WT plants (Fig. 2f). Taken together, these results provide strong evidence that overexpression of *PtrbZIP12* improves drought tolerance in transgenic poplars.

### *PtrbZIP12* enhances ROS scavenging ability

**Figure 3:**
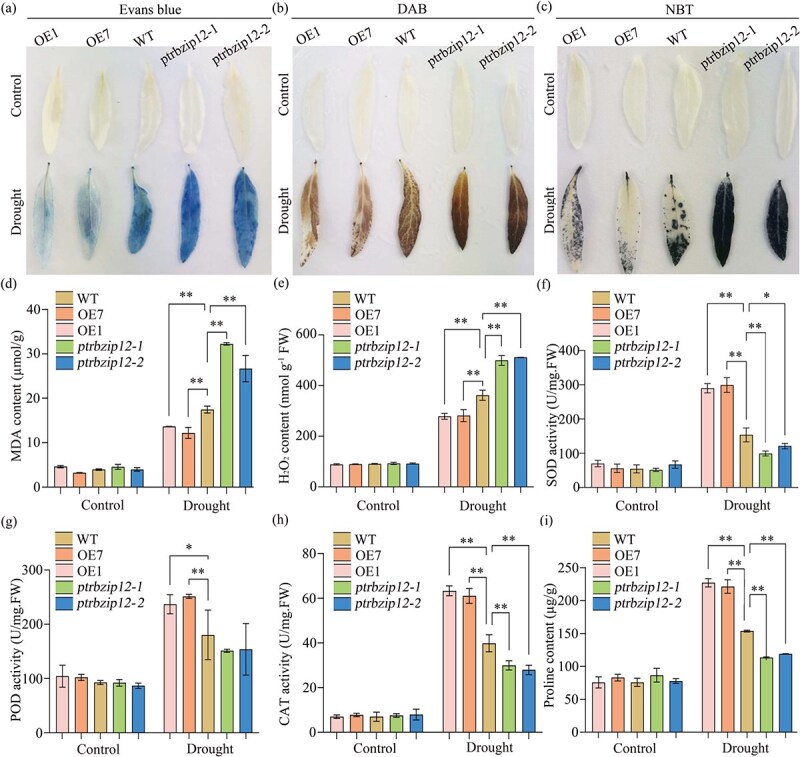
Determination of drought tolerance through physiological and histochemical analyses. (a) Evans blue staining for assessing cell membrane damage. (b) DAB staining for H_2_O_2_ detection. (c) NBT staining for O_2_•^−^ detection. (d) Quantification of MDA content. (e) Measurement of H_2_O_2_ content. (f) SOD activity analysis. (g) POD activity analysis. (h) CAT activity analysis. (i) Proline content analysis. Error bars represent SDs from three biological replicates. Moreover, statistically significant variances are shown as follows: ^*^P < 0.05 and ^**^P < 0.01.

To investigate how the *PtrbZIP12* gene modulates drought tolerance in poplar at the physiological level, we measured key physiological parameters associated with drought stress responses. Under drought conditions, malondialdehyde (MDA) levels in *PtrbZIP12* OE lines were significantly lower than those in WT plants, whereas *ptrbzip12* lines displayed a marked elevation in MDA accumulation (Fig. 3d). These results suggest that the different genotypes experience varying degrees of oxidative damage, with OE plants showing enhanced protection against lipid peroxidation and mutants displaying heightened susceptibility.

To further evaluate cellular integrity under drought stress, cell membrane damage was assessed using Evans blue staining, which revealed more severe membrane injury in *ptrbzip12* plants compared to OE lines, as evidenced by the stronger staining intensity (Fig. 3a). This observation was consistent with the MDA accumulation patterns, reinforcing the conclusion that *PtrbZIP12* plays a protective role in preserving membrane stability during water deficit conditions.

Moreover, the accumulation of ROS was evaluated by histochemical staining. Nitroblue tetrazolium (NBT) was utilized to identify superoxide anion (O_2_•^−^), and 3,3′-diaminobenzidine (DAB) was utilized to identify hydrogen peroxide (H_2_O_2_). Under normal growth conditions, all lines exhibited comparable ROS levels with no significant differences. Under drought conditions, the *PtrbZIP12* mutant plants exhibited the greatest buildup of both O_2_•^−^ and H_2_O_2_, while the OE lines displayed the lowest amounts (Fig. 3b and c). Quantitative measurements of H_2_O_2_ content corroborated the staining results, revealing that drought-stressed *PtrbZIP12* lines accumulated significantly more H_2_O_2_ than the control plants (Fig. 3e), further supporting the role of *PtrbZIP12* in mitigating oxidative stress.

To further elucidate the mechanism by which *PtrbZIP12* contributes to drought tolerance, we investigated whether its OE could improve the scavenging of ROS. Under normal conditions, no notable differences were found in the activities of key antioxidant enzymes, POD, CAT, and SOD, between WT and transgenic plants. In contrast, during drought stress, the OE lines demonstrated significantly increased POD, CAT, and SOD activities relative to WT, while the *PtrbZIP12* mutants exhibited substantially decreased enzyme activities (Fig. 3f–h). These comprehensive physiological analyses indicate that *PtrbZIP12* OE enhances antioxidant capacity, mitigates oxidative damage, and maintains membrane integrity under drought stress, thereby improving the poplar’s drought tolerance.

### *PtrbZIP12* positively regulates proline biosynthesis

As a key osmoregulatory compound in plants, proline not only helps maintain cell membrane structural stability but also functions in scavenging ROS. In this study, we quantified the proline content in all lines before and after drought stress and found that OE plants accumulated markedly higher proline levels than both *ptrbzip12* and WT plants, with the *ptrbzip12* lines showing the lowest content (Fig. 3i). To elucidate the molecular basis of this pattern, we analyzed the expression of two key proline biosynthesis genes, *PtrP5CS1* and *PtrP5CS2*, across all lines. Under drought conditions, both genes were markedly upregulated in OE lines relative to WT, OE, and *ptrbzip12* plants, with the lowest expression observed in *ptrbzip12* line (Fig. S2). This strong correlation between PtrbZIP12 expression and the activation of *PtrP5CS* genes indicates that *PtrbZIP12* positively regulates proline biosynthesis at the transcriptional level, thereby promoting proline accumulation and increasing drought tolerance in transgenic plants.

In summary, *PtrbZIP12* enhances the drought tolerance of transgenic plants by activating the antioxidant enzyme system to scavenge ROS, mitigating membrane damage through reduced lipid peroxidation, and positively regulating proline biosynthesis via the upregulation of key proline biosynthesis genes.

### Screening of *PtrbZIP12* downstream target genes

Based on the functional characterization findings, we explored the molecular regulatory network of *PtrbZIP12* in response to drought stress by pinpointing possible downstream target genes via transcriptome profiling. RNA-seq analysis comparing WT plants and *PtrbZIP12*-OE lines under drought conditions identified 1972 differentially expressed genes (DEGs), comprising 1462 upregulated and 510 downregulated transcripts. A significant portion of these DEGs are established drought-responsive genes, emphasizing *PtrbZIP12*’s pivotal role in controlling essential elements of drought stress signaling pathways.

*PtrbZIP12*-OE plants under drought stress exhibited elevated antioxidant enzyme activities, reduced membrane damage, and enhanced proline accumulation. We focused on gene clusters within the DEGs associated with oxidative stress response and osmoprotection (Fig. S3). Among these, the genes encoding *PtrPOD* and *PtrDHN* showed marked upregulation and were therefore prioritized as the most likely direct downstream targets of PtrbZIP12.

### *PtrDHN* and *PtrPOD* are direct target genes of PtrbZIP12

**Figure 4:**
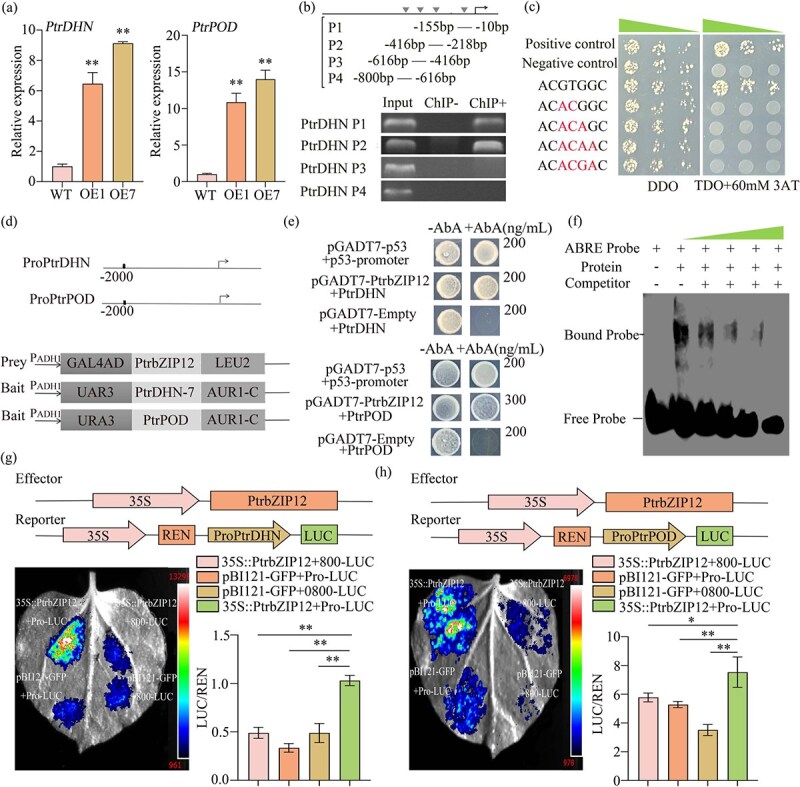
PtrbZIP12 directly activates the promoters of *PtrDHN* and *PtrPOD*. (a) Relative expression levels of *PtrDHN* and *PtrPOD* in *PtrbZIP12*-OE versus WT plants. Data are presented as means ± SD from three biological replicates. (b) Verification of the direct regulatory interaction between *PtrbZIP12* and the *PtrDHN* promoter using ChIP-PCR, in which the promoter region (−800 to −1 bp) was divided into four fragments (P1–P4). ‘Input’ represents chromatin before immunoprecipitation, ‘ChIP-’ indicates immunoprecipitation without antibody, and ‘ChIP+’ denotes immunoprecipitation with anti-GFP antibody. (c) Y1H assay demonstrating *PtrbZIP12* binding to ABRE motifs; controls included p53-HIS2/pGADT7-Rec2-p53 (positive) and pGADT7-Rec2-*PtrbZIP12*/p53-HIS2 (negative). (d and e) Y1H assays showing PtrbZIP12 binding to the promoters of *PtrDHN* and *PtrPOD*, with P53-promoter-AUR1-C and AD-Rec-P53 as positive controls, and AD-empty prey vector with AUR1-C driven by the target gene promoter as negative controls. (f) EMSA confirming *PtrbZIP12*–ABRE binding; lanes: 1, biotin-labeled probe; 2, labeled probe + *PtrbZIP12* protein; 3–5, competition with 10-, 50-, and 100-fold molar excess of unlabeled probe. (g and h) Schematic representation of effector and reporter constructs utilized in the dual-LUC assay, with transient LUC/renillase (REN) coactivation experiments in *N. benthamiana* leaves demonstrating *PtrbZIP12*-mediated activation of *PtrDHN* (g) and *PtrPOD* (h).

To confirm the regulatory connection between *PtrbZIP12* and its putative target genes found by transcriptome analysis, we performed qRT-PCR to measure the expression of *PtrPOD* and *PtrDHN* in *PtrbZIP12*-OE plants. Compared to the WT, the *PtrbZIP12*-OE transgenic lines showed markedly increased expression of both *PtrPOD* and *PtrDHN* (Fig. 4a). Conversely, the two *ptrbzip12* knockout lines exhibited significantly reduced expression of these genes compared to WT under the same drought conditions (Fig. S4). This reciprocal expression pattern validates *PtrbZIP12*’s positive role in activating these stress-responsive genes.

TFs generally regulate the transcription of target genes by binding to particular cis-acting elements in their promoters. To clarify the molecular mechanism behind *PtrbZIP12*-mediated regulation, we used several complementary methods. Bioinformatic analysis revealed ABRE motifs in the promoters of both target genes, and *PtrbZIP12*’s specific binding to these ABRE elements was validated through yeast one-hybrid (Y1H) and electrophoretic mobility shift assays (EMSAs) (Fig. 4c and f). Furthermore, Y1H assays verified the direct protein–DNA interactions between PtrbZIP12 and ABRE-containing promoter fragments of *PtrDHN* and *PtrPOD* (Fig. 4d and e).

To assess whether *PtrbZIP12* directly interacts with the *PtrDHN* promoter *in vivo*, we performed a chromatin immunoprecipitation (ChIP) combined with qPCR (ChIP-qPCR) assay, which verified the association of *PtrbZIP12* with the *PtrDHN* promoter region in plants (Fig. 4b). Furthermore, dual-luciferase (dual-LUC) reporter assays in tobacco leaves demonstrated that PtrbZIP12 significantly activated the expression of both *PtrDHN* and *PtrPOD*. Collectively, these findings provide strong evidence that *PtrDHN* and *PtrPOD* are direct downstream targets positively regulated by PtrbZIP12.

### *PtrDHN* and *PtrPOD* confer drought tolerance to transgenic plants

**Figure 5:**
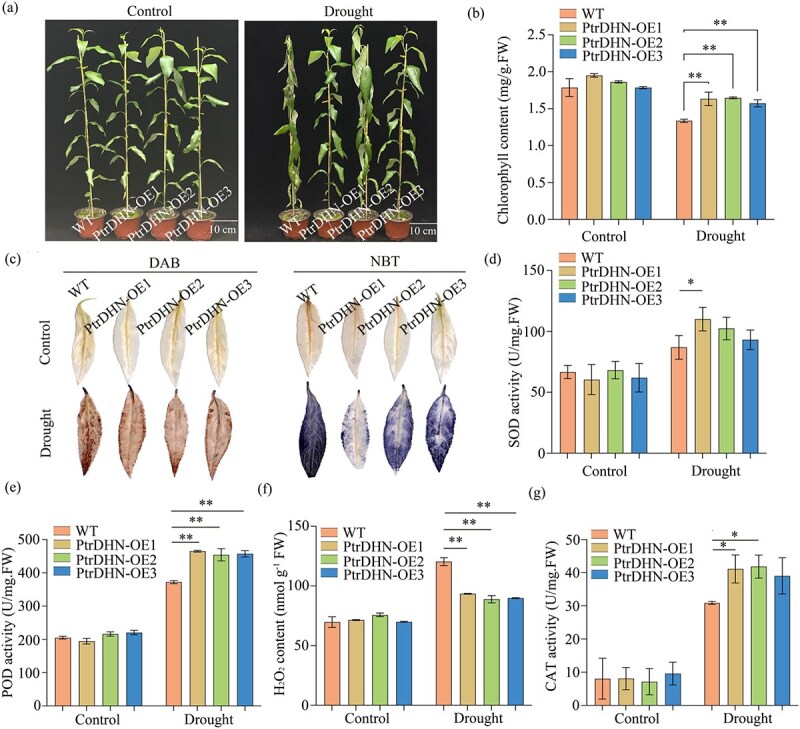
Analysis of drought tolerance mediated by *PtrDHN*. (a) Phenotypic differences among WT and *PtrDHN-OE* lines were assessed under both well-watered (control) and drought-stressed conditions, with the latter induced by a 7-day water-withholding treatment; the scale bar represents 10 cm. (b) Chlorophyll (Chl) content evaluations were conducted. (c) DAB staining and NBT staining. (d) SOD activity; (e) POD activity; (f) H_2_O_2_ content; (g) CAT activity. Error bars represent the SD of three biological replicates (^*^P < 0.05, ^**^P < 0.01).

**Figure 6:**
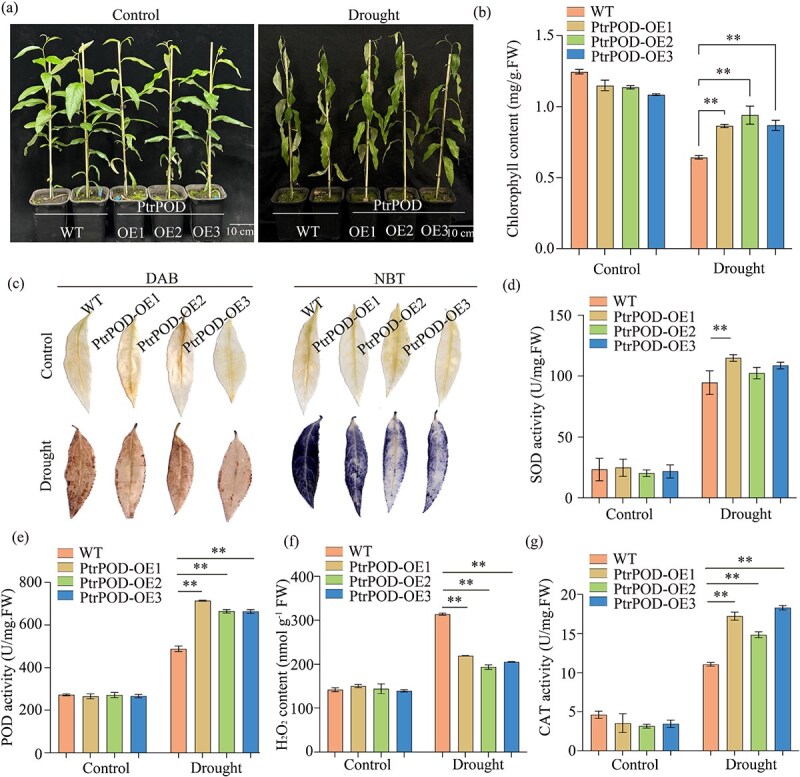
Evaluation of drought tolerance regulated by *PtrPOD*. (a) Phenotypic differences among WT and *PtrPOD-OE* lines were assessed under both well-watered (control) and drought-stressed conditions, the latter induced by a 7-day water-withholding treatment; the scale bar represents 10 cm. (b) Chlorophyll (Chl) content evaluations were conducted. (c) DAB staining and NBT staining. (d) SOD activity; (e) POD activity; (f) H_2_O_2_ content; (g) CAT activity. Error bars represent the SDs from three independent biological replicates (^*^P < 0.05, ^**^P < 0.01).

Through comprehensive molecular and genetic analyses, this study identified *PtrDHN* and *PtrPOD* as direct target genes of *PtrbZIP12*. Bioinformatics analysis combined with expression profiling revealed that both genes are responsive to drought stress (Fig. S5). To functionally characterize their roles, seven *PtrDHN*-OE and three *PtrPOD*-OE transgenic lines were generated for phenotypic evaluation (Fig. S6). Under normal growth conditions, *PtrPOD*-OE and *PtrDHN*-OE plants did not show notable morphological differences compared to WT plants; however, during drought stress, both transgenic lines exhibited significantly enhanced drought tolerance relative to WT (Figs 5a and 6a). Moreover, Similarly, the chlorophyll content was markedly higher in the OE lines relative to WT plants (Figs 5b and 6b). Histochemical staining utilizing DAB and NBT revealed decreased accumulation of O_2_•^−^ and H_2_O_2_ in *PtrPOD*-OE and *PtrDHN*-OE plants compared to WT under drought (Figs 5c and 6c), which aligned with biochemical measurements of H_2_O_2_ levels (Figs 5f and 6f). Additionally, activities of the three main antioxidant enzymes (CAT, POD, and SOD) were markedly elevated in the transgenic lines versus WT during drought stress (Figs 5d, e, g and 6d, e, g). Collectively, these results demonstrate that both *PtrDHN* and *PtrPOD* enhance antioxidant capacity, alleviate oxidative damage, and advance drought tolerance in OE plants, functioning as positive regulators of drought stress resistance.

### PtrbZIP12 and PtrbZIP3 coordinately regulate *PtrDHN* expression

**Figure 7:**
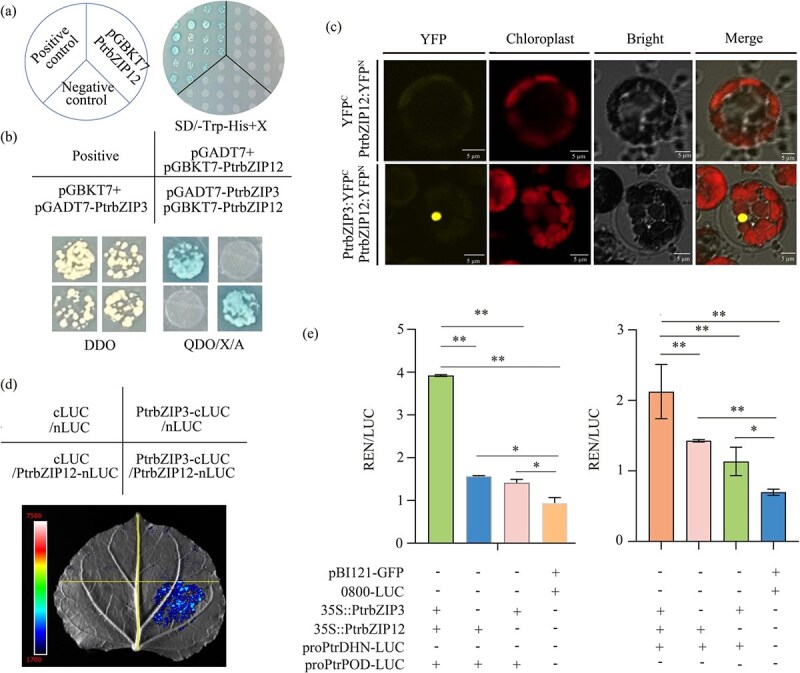
Protein interaction between PtrbZIP12 and PtrbZIP3, which enhances *PtrDHN* transactivation. (a) Transcriptional autoactivation analysis of PtrbZIP12 was conducted, with pGADT7-T + pGBKT7-lam serving as the negative control and pGADT7-T + pGBKT7-p53 as the positive control. (b) Y2H analysis confirmed the interaction between PtrbZIP12 and PtrbZIP3, employing pGADT7-T + pGBKT7-p53 as the positive control and pGADT7-T + pGBKT7-lam as the negative control. (c) BiFC assays demonstrated that PtrbZIP12 interacts with PtrbZIP3 *in vivo*. (d) LCI further verified the PtrbZIP12–PtrbZIP3 interaction through *Agrobacterium*-mediated coexpression in *N. benthamiana* leaves, with luminescence signals detected 48 h after infiltration. (e) Dual-LUC reporter assay showing that PtrbZIP12 and PtrbZIP3 individually activate the *BpDHN/BpPOD* promoter, with coexpression resulting in significantly enhanced relative promoter activity (LUC/REN). Data are presented as mean ± SD (*n* = 6 biological replicates). Statistical significance was determined by Student’s *t* test (^*^P < 0.05, ^**^P < 0.01).

To elucidate the protein interaction network of PtrbZIP12, we systematically investigated its potential interacting partners using multiple complementary approaches. Yeast two-hybrid (Y2H) analysis confirmed that PtrbZIP12 lacks self-activation activity (Fig. 7a), validating its suitability as bait for protein interaction studies. Using the *PtrbZIP12*-pGBKT7 fusion construct as bait to screen a poplar cDNA library via Y2H, we successfully identified PtrbZIP3 as an interacting protein through cloning and sequencing. Y2H assays further verified the interaction between PtrbZIP3 and PtrbZIP12 (Fig. 7b). *In planta* confirmation of this interaction was achieved through bimolecular fluorescence complementation (BiFC) assays, while firefly LUC complementation assays (LCAs) provided quantitative evidence of the interaction (Fig. 7c and d). Considering our previous work showing that *PtrbZIP3* enhances drought resistance in transgenic poplar [54], we next examined the functional consequences of this interaction on downstream gene regulation. Transient coexpression assays in tobacco revealed that coexpression of PtrbZIP12 and PtrbZIP3 significantly increased *PtrDHN* and *PtrPOD* transcript levels compared with expression of either TF alone (Fig. 7e), accompanied by corresponding enhancement of downstream enzymatic activity. PtrbZIP12 interacts with PtrbZIP3 to synergistically enhance the transcriptional activation of downstream target genes.

### Phosphorylation of PtrbZIP12 enhances transcriptional regulation of *PtrDHN* and *PtrPOD*

**Figure 8:**
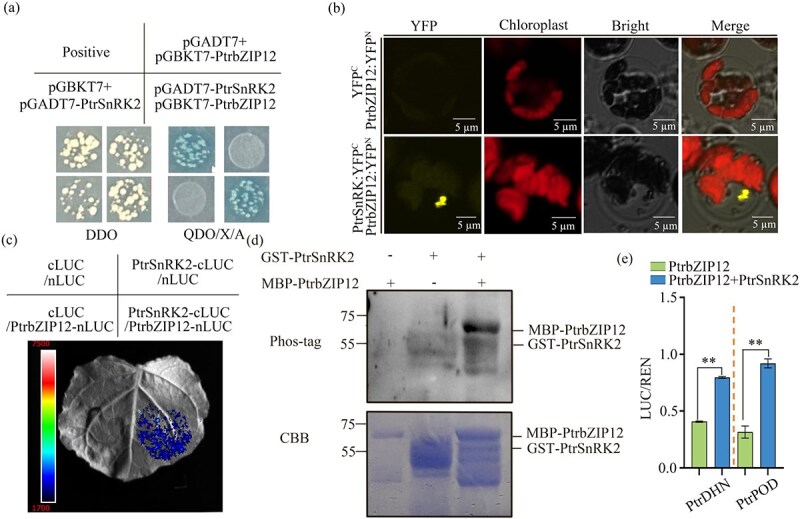
Interaction between PtrbZIP12 and PtrSnRK2. (a) Y2H analysis confirmed the interaction between PtrbZIP12 and PtrSnRK2, with pGADT7-T + pGBKT7-p53 and pGADT7-T + pGBKT7-lam serving as the positive and negative controls, respectively. (b) BiFC assays demonstrated that PtrbZIP12 physically interacts with PtrSnRK2 *in vivo*. (c) Firefly LCI analysis further verified this interaction, in which *Agrobacterium* strains carrying the indicated construct pairs were coexpressed in *N. benthamiana* leaves, and luminescent signals were recorded 48 h after infiltration. (d) *In vitro* phosphorylation assays were performed, with Phos-tag indicating phosphorylation-specific antibody detection and CBB representing Coomassie brilliant blue staining as a loading control. (e) The functional impact of PtrSnRK2–PtrbZIP12 interaction on downstream targets was evaluated using dual-LUC assays, in which LUC/REN ratios for PtrSnRK2 cotransfected with PtrbZIP12 and PtrbZIP12 target genes were normalized against controls containing only PtrbZIP12 and its target genes (^*^P < 0.05, ^**^P < 0.01).

Another interacting protein, PtrSnRK2, was successfully identified through comprehensive Y2H screening, and its interaction with PtrbZIP12 was further validated using Y2H, BiFC, and LUC complementation imaging (LCI) analyses (Fig. 8a–c). Reciprocal Y2H assays confirmed the specific binding between PtrbZIP12 and PtrSnRK2 (Fig. 8a), while BiFC demonstrated their *in planta* interaction, as evidenced by distinct YFP fluorescence signals (Fig. 8b). Firefly LCI further substantiated this interaction (Fig. 8c). An *in vitro* kinase assay was performed to investigate whether PtrSnRK2 acts as a kinase phosphorylating PtrbZIP12, and immunoblotting with an anti-phosphorus antibody confirmed that PtrbZIP12 was phosphorylated by PtrSnRK2 *in vitro*. Moreover, LUC reporter gene assays showed that cotransfection of PtrbZIP12 and PtrSnRK2 in tobacco leaves significantly improved the promoter activity of *PtrDHN* and *PtrPOD*. Collectively, these findings demonstrate that PtrSnRK2 interacts with PtrbZIP12 and acts to activate the expression of its downstream target genes (Fig. 8e).

## Discussion

Drought stress presents a major constraint to plant growth and survival, and with global warming accelerating and arid regions expanding, understanding the regulatory mechanisms underlying drought resistance in woody plants is of critical importance for both global ecological conservation and the sustainable development of the timber industry. As a model tree species and a superior timber cultivar, *P. trichocarpa* provides an ideal system for dissecting drought-responsive transcriptional regulation, offering valuable insights for the genetic improvement of poplar. Building on this context, the present study systematically identifies key bZIP TF family genes and investigates their multilayered roles in drought resistance, encompassing physiological and biochemical adaptations, transcriptional regulatory networks, protein–protein interaction dynamics, and posttranslational modifications. The findings contribute novel molecular biology data that enhance our understanding of stress resistance in forest trees and support future breeding strategies for improved drought tolerance.

### PtrbZIP12 confers drought tolerance via antioxidant activation and proline accumulation

Recent advances have greatly deepened our understanding of plant bZIP proteins in stress responses [39, 40], with growing evidence indicating that bZIP TFs serve as master regulators in numerous abiotic stresses, such as salinity, cold tolerance, and drought [14, 41]. Despite this, the S subfamily of bZIP TFs is still not well characterized. In this study, we identified and examined *PtrbZIP12*, an S subfamily bZIP TF, revealing its function as a new positive regulator in the drought stress response (Fig. 1). Comprehensive physiological analyses showed that *PtrbZIP12* OE lines exhibited elevated proline content and antioxidant enzyme activities, along with reduced oxidative damage markers, compared to WT plants, whereas the opposite trends were observed in *ptrbzip12* knockout plants (Figs 2 and 3). These results are consistent with established stress physiology, in which proline functions as a key osmoprotectant maintaining cellular homeostasis [42], and ROS act as dual-function molecules involved in both stress signaling and cellular damage [43]. Overall, our results offer fresh understanding of the functional importance of S-type bZIP genes in plant adaptation to stress.

### PtrbZIP12 regulates antioxidant and osmotic stress responses through *PtrPOD* and *PtrDHN*

As typical TFs, bZIP proteins exert their biological functions by precisely regulating the expression of downstream effector genes. Previous studies have demonstrated this regulatory paradigm; for example, TaFDL2-1A in wheat mediates ABA-induced growth inhibition by modulating the expression of hormone metabolism genes *TaGH3.2* and *TaGH3.8* via the ACGT core cis-acting element [44], while VlbZIP30 in grapes enhances drought tolerance in transgenic lines by directly activating lignin biosynthesis (*VvPRXN1*) and drought-responsive (*VvNAC17*) genes [45]. In this study, transcriptomic analysis indicated that PtrbZIP12 endows drought tolerance by regulating stress response-related genes. Moreover, through a combination of complementary methods, including EMSA, dual-LUC reporter assays, ChIP-qPCR, and Y1H, we systematically identified and experimentally confirmed *PtrPOD* and *PtrDHN* as direct downstream targets of PtrbZIP12 (Fig. 4).

To alleviate oxidative damage triggered by drought stress, plants regulate ROS balance by adjusting the antioxidant enzymes’ activities, thus diminishing or removing excess ROS buildup [46–48]. Among these enzymes, POD plays a vital role in plant drought stress responses [49–51]. In our study, *PtrPOD*, identified as a direct target of PtrbZIP12, was shown to alleviate ROS toxicity, protect cell membrane integrity, and reduce oxidative stress, ultimately enhancing drought resistance in transgenic poplar. These results suggest that activation of *PtrPOD* by PtrbZIP12 forms a vital part of the plant’s defense system against drought stress. The activation of dehydrin genes by bZIP TFs during drought stress serves as a crucial node in plant adaptive mechanisms. Acting as key mediators of ABA signaling, bZIP proteins, especially those from the AREB/ABF subfamily, bind directly to ABREs located in dehydrin gene promoters, thus inducing transcriptional upregulation under water deficit conditions [52, 53]. In this study, we showed that PtrbZIP12 specifically attaches to the ABRE motif in the promoter region of the dehydrin gene *PtrDHN*, significantly boosting its transcriptional activity (Fig. 5), consistent with earlier reports. Furthermore, *PtrDHN* OE in transgenic plants led to substantial increases in the activities of CAT, POD, and SOD (Fig. 6), suggesting that dehydrins, as highly hydrophilic proteins, aid drought tolerance through osmotic regulation and oxidative stress alleviation. These results further substantiate the pivotal role of the PtrbZIP12–*PtrDHN* regulatory module in orchestrating drought stress responses.

This study outlines a regulatory cascade from TF to downstream effectors, elucidating how *PtrbZIP12* orchestrates multiple drought defense mechanisms in poplar at the molecular level.

### Complex formation enhances PtrbZIP12-mediated transcriptional regulation under drought stress

To clarify the molecular mechanism behind PtrbZIP12-mediated drought resistance, we explored its protein interaction network. Employing a combination of Y2H, LCI, and BiFC assays, we confirmed that PtrbZIP12 interacts with PtrbZIP3 within the nucleus (Fig. 7). PtrbZIP3, a nuclear-localized TF belonging to subfamily A, possesses established transcriptional activation activity and has been reported as a positive regulator of drought stress responses [54]. Typically, TFs form complexes with other proteins to jointly regulate specific gene expression. Examples include the interaction between DgbZIP3 and DgbZIP2, which boosts *DgPOD* expression to improve cold stress tolerance in chrysanthemum [55], and the formation of bZIP71–bZIP73 heterodimers that substantially enhance grain yield and seed-setting rate under natural cold stress [24]. Our findings reveal a novel cross-subfamily interaction between the S-subfamily protein PtrbZIP12 and the A-subfamily protein PtrbZIP3. Functional characterization showed that the PtrbZIP12–PtrbZIP3 heterodimer exhibits markedly stronger transactivation of the *PtrDHN/PtrPOD* promoter than either monomer alone (Fig. 7), indicating that heterodimer composition functions as a molecular rheostat to fine-tune stress-responsive gene expression. This PtrbZIP12–PtrbZIP3 module exemplifies how plants integrate multiple stress signals through protein–protein interactions to optimize adaptive responses, providing new perspectives for engineering drought resistance in perennial species.

### PtrSnRK2 regulates PtrbZIP12 through phosphorylation

SnRK2 kinases are central regulators of abscisic acid (ABA) signaling and drought stress responses in plants. Beyond their well-established role in posttranslational phosphorylation, accumulating evidence indicates that the expression of SnRK2 genes themselves is often induced by osmotic stress, underscoring their pivotal position in stress adaptation networks. For instance, *TaSnRK2.9* overexpression plants exhibited enhanced tolerance to drought and high salinity in young seedlings and mature plants [56]. Similarly, in apple, *MdSnRK2.10* is upregulated by drought and ABA treatments, conferring improved drought resistance in both transgenic *apple* and *Arabidopsis* [57]. In tea plant, *CsSnRK2.5* is a PEG_6000_-induced *SnRK2* gene; its overexpression enhances drought tolerance in *Arabidopsis* [58].These findings highlight the conserved and essential role of SnRK2 kinases in engineering abiotic stress tolerance across diverse crop species.

Our investigation revealed that PtrbZIP12 undergoes posttranslational modification via phosphorylation by PtrSnRK2, adding an additional regulatory layer to drought stress responses. This observation aligns with increasing evidence of SnRK-driven regulation of stress-responsive TFs. For example, CaMEKK23 acts as a positive regulator in ABA-mediated drought responses in chili plants, with its phosphorylation regulated by CaPP2Cs and CaSnRK2.6, establishing it as a central element of ABA signaling [59]. Additionally, phosphorylation of the TF bZIP39 by SnRK1 kinase controls xylitol metabolism in apple [60]. Such interactions between phosphorylation and other posttranslational modifications underscore the regulatory complexity of SnRK2 in stress adaptation. In our study, LUC reporter assays demonstrated that cotransfection of PtrbZIP12 and PtrSnRK2 in tobacco leaves significantly increased the promoter activities of *PtrDHN* and *PtrPOD* (Fig. 8). Collectively, these findings indicate that the PtrSnRK2–PtrbZIP12 module constitutes a key signaling node that integrates kinase-mediated phosphorylation with transcriptional regulation to enhance drought stress responses.

Collectively, these findings demonstrate that drought stress responses mediated by *PtrbZIP12* in poplar are orchestrated through multilayered regulatory mechanisms, encompassing transcriptional and posttranslational control, signal integration via convergence of kinase cascades and TF networks, and dynamic modulation through phosphorylation-dependent activity tuning.

## Conclusion

**Figure 9:**
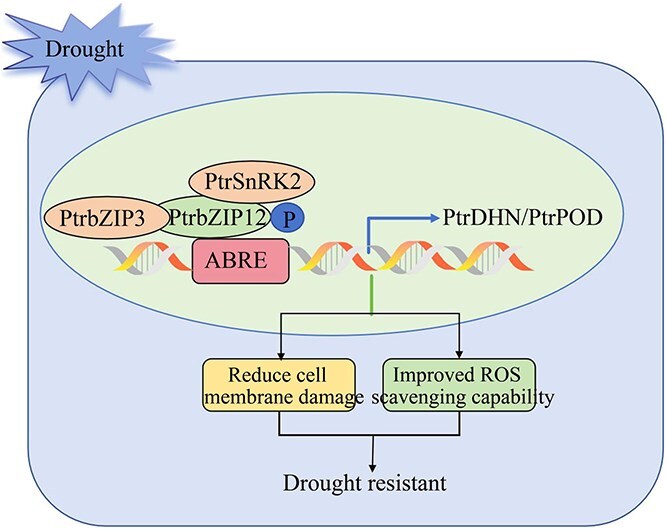
Schematic diagram of *PtrbZIP12* regulating the drought resistance mechanism of *P. trichocarpa.*

In this research, we identified *PtrbZIP12*, a bZIP TF belonging to the S subfamily, which shows strong responsiveness to drought stress, and we thoroughly investigated its functional role under these conditions. Our findings demonstrate that *PtrbZIP12* significantly enhances drought tolerance in transgenic poplar, thereby underscoring its pivotal role in stress adaptation. Further investigation into its regulatory network revealed that PtrbZIP12 confers drought tolerance by functioning as an upstream regulator of *PtrDHN* and *PtrPOD*, thereby modulating critical downstream protective mechanisms. Moreover, we elucidated the phosphorylation-dependent mechanism underlying *PtrbZIP12*-mediated drought tolerance, providing novel insights into the signal transduction processes that link stress perception to transcriptional reprogramming in poplar (Fig. 9).

## Materials and methods

### Plant material and drought treatment

*Populus trichocarpa* was used as the primary plant material in this study. Seedlings were initially cultivated on solid plant medium (WPM). Stem nodes (second to fourth) from 1-month-old, *in vitro*-grown seedlings were excised and used as explants for AMT. For experiments conducted in soil, 1-month-old seedlings grown *in vitro* were transferred into pots filled with soil amended with charcoal. The plants were kept in a growth chamber at 21°C–25°C under a 16-h light/8-h dark cycle, with light intensity ranging from 200 to 300 μmol m^−2^ s^−1^ and relative humidity controlled between 60% and 80%.

Following 1 month of growth in soil, seedlings underwent drought stress treatment using PEG_6000_ for durations of 6, 12, 24, or 48 h, with untreated, well-watered plants serving as controls. Tissues were harvested at each time point for subsequent analyses. For phenotypic evaluation under extended drought, 3-month-old WT and transgenic seedlings grown in soil were exposed to natural drought stress by withholding water for 7 days. Throughout the treatment, phenotypic alterations were regularly observed.

### RNA extraction and qRT-PCR

Total RNA was extracted from the collected samples utilizing an RNA extraction kit (Biomarker, RK02010) according to the manufacturer’s guidelines. RNA quality was evaluated by 1% agarose gel electrophoresis, showing an approximate 28S:18S rRNA ratio of 2:1. cDNA synthesis was carried out using the Takara Reverse Transcription Kit. Gene-specific primers were designed via Primer-BLAST (NCBI). Each qPCR reaction mixture consisted of 10-μl SYBR Green Master Mix (Bio-Rad), 0.2 μM of each primer, and 50-ng cDNA, totaling 20 μl, and was run on a Bio-Rad CFX96 system. Relative expression levels were determined by the 2^−ΔΔCt^ method, normalized to GAPDH. Results from three independent replicates were expressed as mean ± SD, and statistical analysis was performed using Student’s *t* test with GraphPad Prism 9.0.

### GUS staining

The *PtrbZIP12* gene promoter plant expression vector (pCAMBIA1301-GUS) was constructed, and *ProPtrbZIP12*::GUS transgenic material was generated via AMT. *ProPtrbZIP12*::GUS plants aged 1 month were treated with 20% PEG_6000_ for 24 h, with untreated plants used as controls. After treatment, the plants were promptly submerged in GUS staining buffer, vacuum infiltrated to improve dye penetration, and incubated in darkness at 37°C for 12 to 24 h. Decolorization was achieved by washing with 70% ethanol until the background was clear. Finally, the samples were transferred to 70% glycerol and examined under a microscope for imaging.

### LUC reporter gene assay

For LUC reporter assays, the *PtrPOD* and *PtrDHN* promoter sequences were inserted into the pGreen0800 vector using SalI and KpnI restriction enzymes. The *PtrbZIP12* promoter (1500-bp upstream of the ATG start site) along with the pBI121-*PtrbZIP2*-GFP vector was transformed into *Agrobacterium tumefaciens* strain GV3101. In transient expression experiments, promoter::LUC reporter constructs were generated, and *Agrobacterium* cultures harboring these constructs were infiltrated into *tobacco* leaves, with empty vectors used as controls. LUC activity was quantified following the assay kit’s instructions.

To evaluate the drought responsiveness of the *PtrbZIP2* promoter, a 1500-bp fragment was cloned into the pGreen0800 vector utilizing KpnI and SalI restriction sites. Transient expression assays were carried out in tobacco leaves, and after 48 h, the plants were exposed to 20% PEG_6000_ for 15 min. LUC reporter gene activity was measured according to the kit instructions, and leaf fluorescence was detected by infiltrating d-fluorescein solution into the leaves, followed by imaging using the Xenogen system.

### Subcellular localization and transcription activation activity analysis

To study the subcellular localization of PtrbZIP12, the pBI121-*PtrbZIP2*-GFP fusion expression vector was generated. Localization analysis was performed using laser confocal microscopy (LSM700, Zeiss, Jena, Germany) following *Agrobacterium rhizogenes* GV3101-mediated transient transformation of *N. benthamiana* epidermal cells. Additionally, the *PtrbZIP12*-pGBKT7 fusion expression vector was generated using Infusion cloning technology. The resulting construct was transformed into Y2H yeast receptor cells following the protocol of the Takara Yeast Transformation Kit. The transcriptional activity of PtrbZIP2 was then assessed using SD/-Trp-His/X-α-Gal screening.

### *PtrbZIP12* OE and preparation of mutant material

The OE vector for *PtrbZIP12* was built using the identical backbone as that employed for the subcellular localization vector. For gene knockout, two specific CRISPR/Cas9 targets were designed based on the *PtrbZIP12* coding sequence and targeting criteria. Using pCBC-DT1T2 as a template, target fragments were amplified with four primers (-F0/-R0 and -BsF/-BsR) and subsequently cloned into the pHSE401 gene editing vector through gel purification, restriction enzyme digestion, and T4 ligase-mediated ligation. Following established protocols, stable *Agrobacterium*-mediated transformation was performed using *P. trichocarpa* stem segments as explants [54]. The pBI121-*PtrbZIP12*-GFP and the pHSE401 CRISPR/Cas9 editing vector were introduced into plants. After selection on kanamycin and hygromycin, we successfully obtained *PtrbZIP12* gene mutants (*ptrbzip12*) and 35S::*PtrbZIP12* lines.

### Physiological and biochemical index analysis

Chlorophyll and MDA levels were determined based on the method detailed by Zang *et al.* Staining with Evans blue, nitroblue NBT, and 3,3′-DAB was carried out as outlined by Zang *et al.* [61]. Activities of CAT, SOD, and POD, along with H_2_O_2_ content and proline concentration, were measured utilizing commercial assay kits from Nanjing Bioengineering Institute. Each sample included at least 10 plants, and all assays were repeated three times to confirm biological reproducibility.

### Electrophoretic mobility shift assay

The *PtrbZIP12* coding sequence was inserted into the pMAL-c5x vector to create the recombinant plasmid pMAL-c5x-*PtrbZIP12*, which was subsequently introduced into competent *Escherichia coli* ER2523 cells. Protein expression was triggered by the addition of 0.5 mM isopropyl-β-d-thiogalactopyranoside. The expressed fusion proteins were purified via affinity chromatography utilizing the maltose-binding protein tag with Ni-NTA™ resin (Shanghai Sangong Bioengineering). Specific biotin-labeled DNA probes targeting the promoter region of the gene were synthesized by Sangon Biotech Co. Using a chemiluminescence detection kit (Biyun Tian, GS009), EMSA was conducted. Following the binding reaction, samples were subjected to electrophoresis, transferred to membranes, and cross-linked. Probe migration was detected by chemiluminescence (Biyun Tian, P0018S) and imaged using a Tanon-5200 imaging system.

### Yeast one-hybrid

PCR-amplified promoter fragments of *PtrPOD* and *PtrDHN* were cloned into the pAbAi vector positioned upstream of the aureobasidin A (AbA) resistance gene. The coding sequence of *PtrbZIP12* was introduced into the pGADT7 vector to produce a prey plasmid expressing the TF fused with the GAL4 activation domain (TF-AD). The bait strains (Y1HGold-pAbAi) were cotransformed with the prey plasmid using the PEG/LiAc method. Transformants were grown on SD/-Ura/-Leu medium containing AbA and incubated for 3 to 5 days at 30°C. Colonies showing resistance to AbA were then streaked for confirmation.

### Yeast two-hybrid

The *PtrbZIP12*-pGBKT7 construct was generated and introduced into yeast receptor cells per the manufacturer’s guidelines. Single colonies containing *PtrbZIP12*-pGBKT7 were grown in SD/-Trp liquid medium until the optical density at 600 nm (OD_600_) reached about 0.8. These yeast cells were then mated with a Mauve poplar cDNA yeast expression library. After observing the formation of mating structures, initial screening was conducted on DDO/X/AbA plates, followed by rescreening on QDO/X/AbA plates. Blue-spotted clones from the final screening were sequenced and analyzed to identify proteins interacting with *PtrbZIP12*.

### RNA-Seq and ChIP analysis

To analyze DEGs between WT and *PtrbZIP12*-OE plants, RNA-seq was conducted with three independent biological replicates. Sequencing was conducted by UWTSD. Concurrently, 35S::*PtrbZIP12* plants were subjected to ChIP assays using a commercial kit (Millipore). Tissue samples were fixed in a solution comprising 10 mM Tris–HCl (pH 8.0), 1% (w/v) formaldehyde, 0.4 M sucrose, 1 μg/ml protease inhibitors, and 1 mM phenylmethylsulfonyl fluoride. Following vacuum infiltration for cross-linking, the reaction was stopped by treating samples with 125 mM glycine for 5 min. Chromatin was then fragmented using a Covaris sonicator and subjected to immunoprecipitation with an anti-GFP antibody. The isolated DNA was examined by ChIP-PCR with gene-specific primers.

### Protein interaction

Coding sequences of candidate proteins PtrbZIP3 and PtrbZIP12 were cloned into vectors carrying the N-terminus and C-terminus of YFP, respectively. An empty vector was used as the negative control. These recombinant plasmids were transiently introduced into protoplasts using PEG_4000_-mediated transformation according to standard procedures. After incubation in darkness for 36–48 h at 25°C, fluorescence was detected via confocal microscopy with excitation at 514 nm and emission recorded between 527 and 550 nm (YFP channel).

For the LCA conducted in *N. benthamiana* leaves, *PtrbZIP12*’s coding sequence was inserted into the pCAMBIA1300-nLUC vector at the KpnI and SalI restriction sites, whereas the full-length PtrbZIP3 sequence was cloned into the identical sites of the pCAMBIA1300-cLUC vector. *Agrobacterium tumefaciens* strain GV3101 cultures (OD_600_ = 0.8) harboring these constructs were infiltrated into tobacco leaves using the standard infiltration method. LUC signals were detected as previously described.

### *In vitro* phosphorylation assay

PtrbZIP12 and PtrSnRK2 proteins were purified using prokaryotic expression systems for use in EMSA. At room temperature, 1 μg of PtrSnRK2 was incubated with 0.5 μg of either WT or mutated PtrbZIP12 in kinase reaction buffer (40 mM Tris–HCl, pH 7.4; 1 mM MgCl_2_; 10 μM ATP) for 30 min. Phosphorylation of *PtrbZIP12* by PtrSnRK2 was subsequently detected by Western blot analysis using Phos-biotin detection reagents (APExBIO, Houston, TX, USA). All the primers used are shown in Table S2.

### Accession numbers

PtrbZIP12 (Potri.014G007100);

PtrbZIP3 (Potri.004G140600);

PtrPOD (Potri.008G022232);

PtrSnRK2 (Potri.007G096400);

PtrDHN (Potri.003G138700).

## Supplementary Material

Web_Material_uhag034
