# Supplementary material for: PtrbZIP12 improves drought resistance in Populus trichocarpa by directly targeting PtrDHN and PtrPOD
Source: Hortic Res. 2026 Feb 5;13(5):uhag034. doi: 10.1093/hr/uhag034 (PMC13148172; doi:10.1093/hr/uhag034)
Supplement: Web_Material_uhag034 [file web_material_uhag034.zip › Supplementary Figure.pdf]

Supplementary Figure S1.

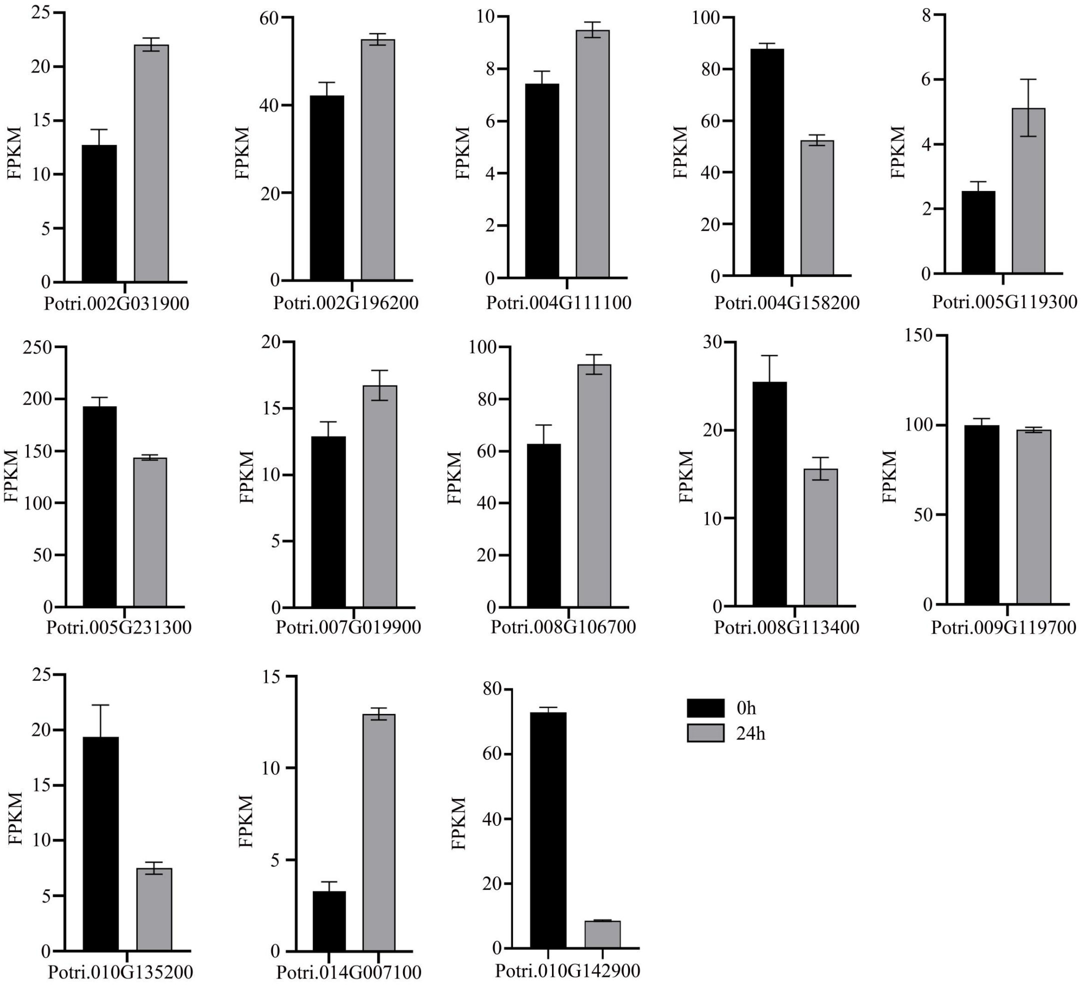

Figure S1. Expression Analysis of PtrS-bZIP Subfamily Members in *Populus trichocarpa.*

Supplementary Figure S2.

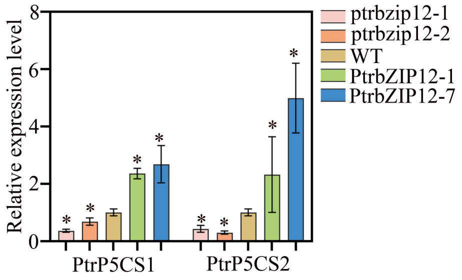

Figure S2. Expression of *P5CS* gene in OE, *ptrbzip12* and WT plants under drought. Poplar growth phenotypes were monitored under normal conditions and drought stress for 90 days, with control plants receiving regular watering and drought-stressed plants subjected to 7 days without water. Each experimental replicate consisted of 5-10 plantlets. Data are presented as means ± SD ($n \geq 3$). Asterisks denote significant differences versus the control (one-way ANOVA with Tukey's test). *P<0.05.

Supplementary Figure S3.

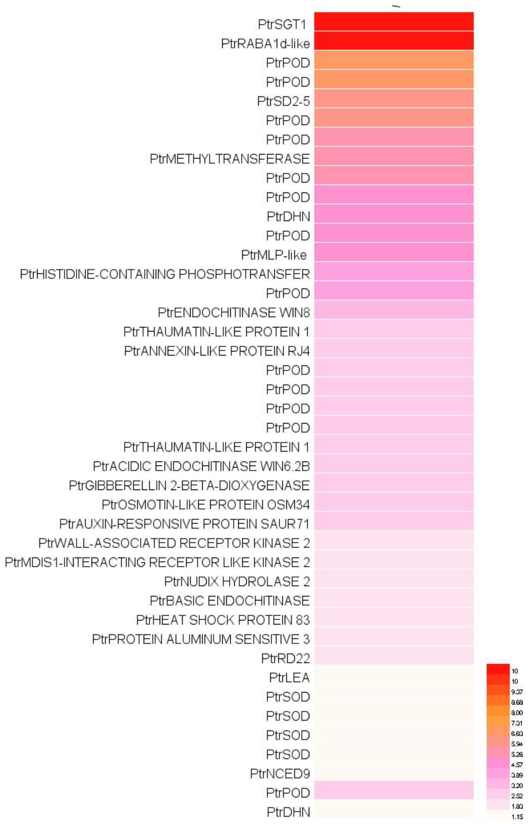

Figure S3. Differential expression gene heat map.

Supplementary Figure S4.

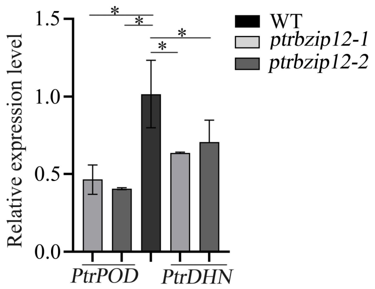

Figure S4. Relative expression levels of *PtrDHN* and *PtrPOD* in *ptrbzip12* and WT plants. Data are presented as means ± SD (n ≥ 3). Asterisks denote significant differences versus the control (one-way ANOVA with Tukey's test)(*$P < 0.05$).

Supplementary Figure S5.

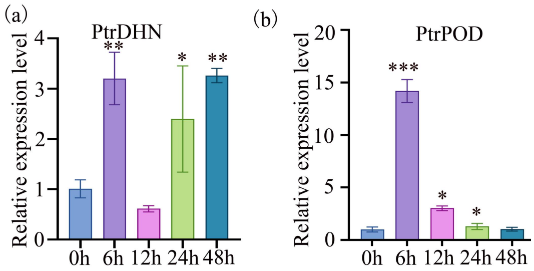

Figure S5. Expression characteristics of *PtrDHN* and *PtrPOD*. (a) Expression patterns of *PtrDHN* under 20% PEG_{6000} treatment. (b) Expression patterns of *PtrPOD* under 20% PEG_{6000} treatment. Data are presented as the mean ± SD (n = 6, combining three biological replicates with three technical replicates each). Asterisks denote significant differences versus the control (one-way ANOVA with Tukey's test) (**P* < 0.05, ***P* < 0.01, ****P*< 0.001).

Supplementary Figure S6.

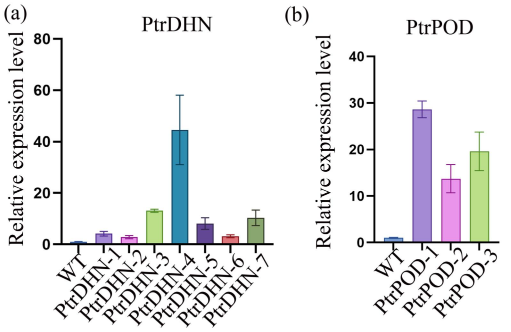

Figure S6. qRT-PCR analysis of the relative expression of *PtrDHN* (a) and *PtrPOD* (b) in the OE lines. Data are presented as the mean ± SD (n = 6, combining three biological replicates with three technical replicates each).
