# Supplementary figures and images for: PtrbZIP12 improves drought resistance in Populus trichocarpa by directly targeting PtrDHN and PtrPOD
Source: Hortic Res. 2026 Feb 5;13(5):uhag034. doi: 10.1093/hr/uhag034 (PMC13148172; doi:10.1093/hr/uhag034)

Supplementary Figure S1.

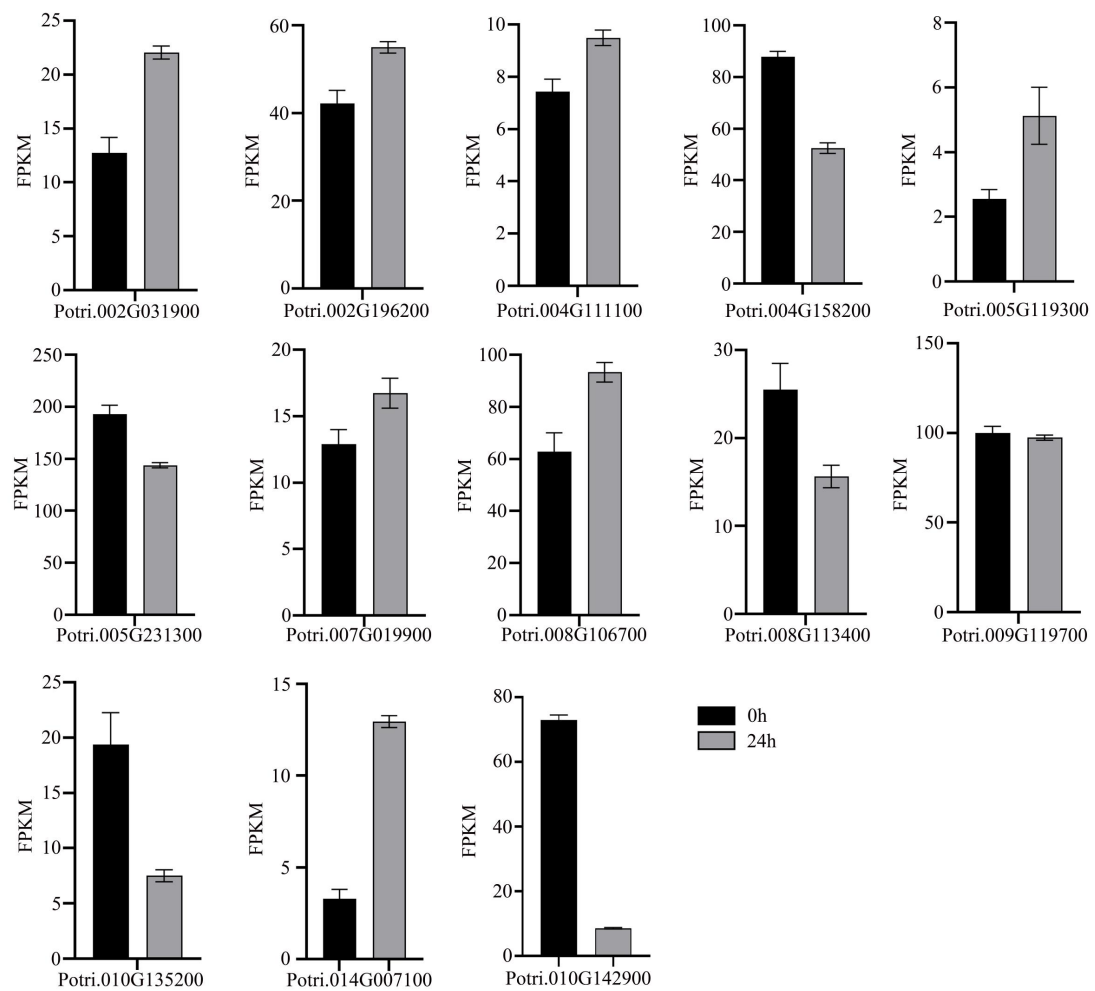

Supplement: Web_Material_uhag034 [file web_material_uhag034.zip › Supplementary Figure_1.pdf]

Supplementary Figure S2.

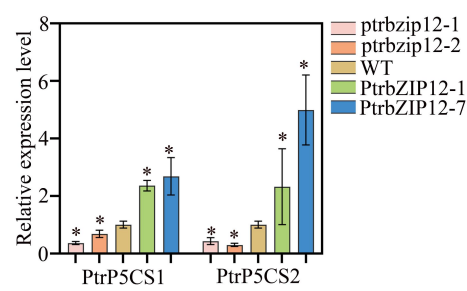

Supplement: Web_Material_uhag034 [file web_material_uhag034.zip › Supplementary Figure_2.pdf]

Supplementary Figure S3.

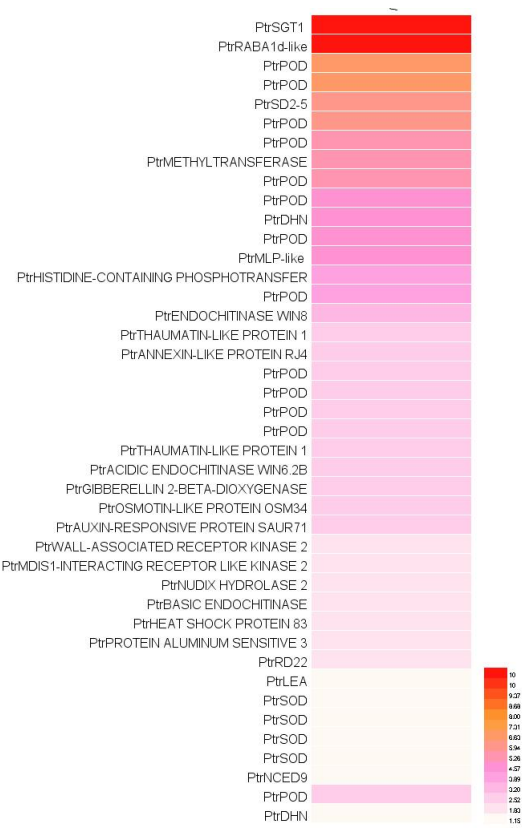

Supplement: Web_Material_uhag034 [file web_material_uhag034.zip › Supplementary Figure_3.pdf]

Supplementary Figure S4.

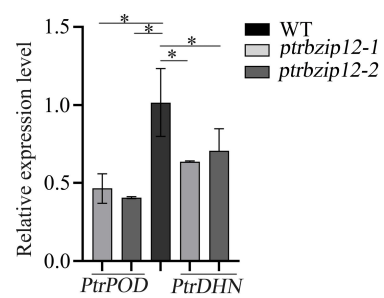

Supplement: Web_Material_uhag034 [file web_material_uhag034.zip › Supplementary Figure_4.pdf]

Supplementary Figure S5.

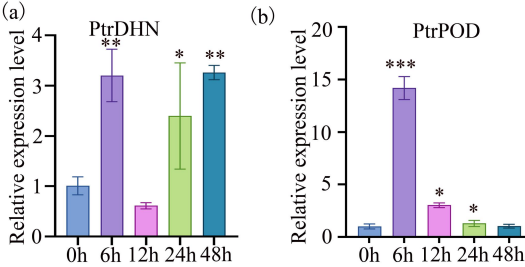

Supplement: Web_Material_uhag034 [file web_material_uhag034.zip › Supplementary Figure_5.pdf]

Supplementary Figure S6.

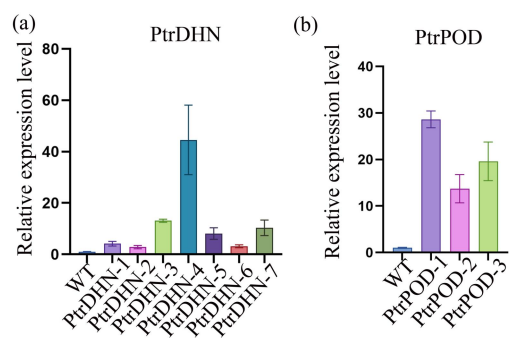

Supplement: Web_Material_uhag034 [file web_material_uhag034.zip › Supplementary Figure_6.pdf]
